# Supplementary material for: Superlative mechanical energy absorbing efficiency discovered through self-driving lab-human partnership
Source: Nat Commun. 2024 May 21;15:4290. doi: 10.1038/s41467-024-48534-4 (PMC11109101; doi:10.1038/s41467-024-48534-4)
Supplement: Supplementary file 1 — Supplementary Information [file 41467_2024_48534_MOESM1_ESM.pdf]

**Supplementary Information**

# Superlative Mechanical Energy Absorbing Efficiency Discovered through Self-Driving Lab-Human Partnership

Kelsey L. Snapp[1], Benjamin Verdier[2], Aldair E. Gongora[1], Samuel Silverman[2], Adedire D. Adesiji[1], Elise F. Morgan[1,3,4], Timothy J. Lawton[5], Emily Whiting[2], and Keith A. Brown[1,3,6]

1 Department of Mechanical Engineering, Boston University, Boston, MA, USA

2 Department of Computer Science, Boston University, Boston, MA, USA

3 Division of Materials Science & Engineering, Boston University, Boston, MA, USA

4 Department of Biomedical Engineering, Boston University, Boston, MA, USA

5 Soldier Protection Directorate, US Army Combat Capabilities Development Command Soldier Center, Natick, MA, USA

6 Physics Department, Boston University, Boston, MA, USA

**Table of Contents**

1. Definition and Records of Mechanical Energy Absorbing Efficiency 2
2. Process for Converting Force-Displacement into Stress-Strain 3
3. Defining a Generalized Cylindrical Shell 4
4. Polymers under Consideration 6
5. The Bayesian Experimental Autonomous Researcher 7
    5.1 Select Experiment 7
    5.2 Generate G-code 11
    5.3 Begin Experiment 12
    5.4 Weigh Component 12
    5.5 Test Component 13
    5.6 Process Results 13
    5.7 Maintenance 14
6. Details of the Experimental Campaign 17
7. Post-Analysis of the Campaign 20
    7.1 Comparison of the Iroko and Willow Designs 20
    7.2 Material-Dependent Performance Envelope 21
    7.3 Statistical Analysis of the Superlative Design 22
8. Supplemental References 24

## Supplementary Note 1. Definition and Records of Mechanical Energy Absorbing Efficiency

The energy absorbing efficiency $K_s$ of a material or structure in compression can be calculated by dividing the amount of energy absorbed before surpassing a stress threshold $\sigma_t$ by the maximum energy that could be absorbed below that threshold, i.e. compressing to an engineering strain of 1 while maintaining an engineering stress $\sigma = \sigma_t$ (Figure 1a). Equivalently, $K_s$ can also be calculated directly from force-displacement data by calculating the amount of energy absorbed before surpassing a force threshold by the maximum amount of energy that could be absorbed below that threshold, *i.e.* compressing until the displacement of the component equals its initial height while maintaining a force equal to the threshold force. For most structures, $K_s$ reaches its maximum energy absorbing efficiency $K_s^*$ at an optimum threshold stress $\sigma_t^*$. This single point, ($\sigma_t^*$, $K_s^*$) can be used to describe the ideal operating performance of a structure or material. To illustrate common and superlative values of $K_s^*$, a summary of literature values is shown graphically in Figure S1. The sources of these points are given in Table S1. Values of $\sigma_t^*$ and $K_s^*$ not directly reported were computed based on reported force-displacement or stress-strain plots. Included on this plot are two values taken from this work.

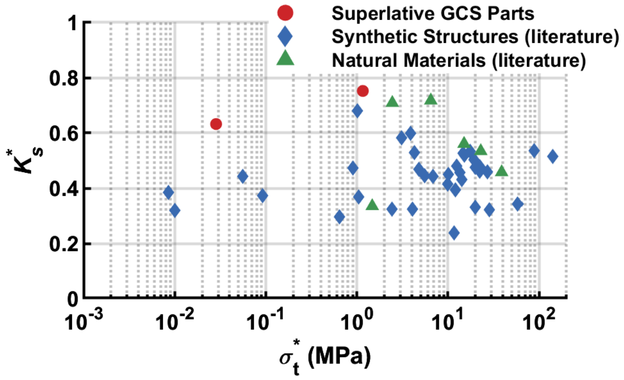


**Fig. S1 | Common and superlative structures and materials**. Synthetic structures (blue diamond) and natural materials (green triangle) gathered from literature, with superlative plastic and hyperelastic generalized cylindrical shells (GCS-this study) components (red circle).

| $\sigma_t^*$ (MPa) | $K_s^*$ (%) | Material origin | Reference |
|---|---|---|---|
| $8.50\times10^{-3}$ | 38.5 | Synthetic | https://doi.org/10.1016/j.matdes.2017.11.037 |
| $1.00\times10^{-2}$ | 32.0 | Synthetic | https://doi.org/10.1016/j.matdes.2017.11.037 |
| $2.85\times10^{-2}$ | 63.2 | Synthetic | This work – ADTS ID 22335 |
| $5.55\times10^{-2}$ | 44.4 | Synthetic | https://doi.org/10.1016/j.matdes.2017.11.037 |
| $9.17\times10^{-2}$ | 37.4 | Synthetic | https://doi.org/10.1016/j.matdes.2017.11.037 |
| $6.47\times10^{-1}$ | 29.7 | Synthetic | https://doi.org/10.1002/admt.201800419 |
| $9.01\times10^{-1}$ | 47.3 | Synthetic | https://doi.org/10.1002/admt.201800419 |
| 1.01 | 68.1 | Synthetic | https://doi.org/10.1177/0021955X06063519 |
| 1.04 | 36.9 | Synthetic | https://doi.org/10.1016/j.actamat.2004.05.039 |
| 1.16 | 75.2 | Synthetic | This work – ADTS ID 21285 |
| 1.47 | 33.5 | Natural | https://doi.org/10.1016/j.jmbbm.2019.103603 |
| 2.42 | 32.3 | Synthetic | https://doi.org/10.1177/0021955X06063519 |
| 2.45 | 70.9 | Natural | https://doi.org/10.1016/S0167-6636(02)00268-5 |
| 3.08 | 58.2 | Synthetic | https://doi.org/10.1016/j.msea.2004.03.051 |

| | | | |
|---|---|---|---|
| 3.89 | 59.8 | Synthetic | https://doi.org/10.1177/0021955X06063519 |
| 4.07 | 32.4 | Synthetic | https://doi.org/10.1177/0021955X06063519 |
| 4.30 | 52.9 | Synthetic | https://doi.org/10.1016/j.msea.2004.03.051 |
| 4.76 | 46.8 | Synthetic | https://doi.org/10.1016/j.actamat.2004.05.039 |
| 5.53 | 44.5 | Synthetic | https://doi.org/10.1016/j.ijimpeng.2010.03.007 |
| 6.47 | 71.8 | Natural | https://doi.org/10.1016/S0167-6636(02)00268-5 |
| 6.88 | 44.2 | Synthetic | https://doi.org/10.1177/0021955X06063519 |
| 9.99 | 41.5 | Synthetic | https://doi.org/10.2140/jomms.2013.8.65 |
| $1.01\times10^1$ | 45.0 | Synthetic | https://doi.org/10.1016/j.actamat.2004.05.039 |
| $1.17\times10^1$ | 23.8 | Synthetic | https://doi.org/10.1002/admt.201800419 |
| $1.21\times10^1$ | 39.4 | Synthetic | https://doi.org/10.1177/0731684419868018 |
| $1.24\times10^1$ | 47.9 | Synthetic | https://doi.org/10.1177/0731684419868018 |
| $1.34\times10^1$ | 45.6 | Synthetic | https://doi.org/10.1177/0731684419868018 |
| $1.42\times10^1$ | 43.2 | Synthetic | https://doi.org/10.1177/0731684419868018 |
| $1.46\times10^1$ | 52.8 | Synthetic | https://doi.org/10.1177/0731684419868018 |
| $1.50\times10^1$ | 56.1 | Natural | https://doi.org/10.1016/S0167-6636(02)00268-5 |
| $1.52\times10^1$ | 51.9 | Synthetic | https://doi.org/10.1177/0731684419868018 |
| $1.74\times10^1$ | 53.3 | Synthetic | https://doi.org/10.1177/0731684419868018 |
| $1.92\times10^1$ | 50.3 | Synthetic | https://doi.org/10.1177/0731684419868018 |
| $1.98\times10^1$ | 47.6 | Synthetic | https://doi.org/10.1177/0731684419868018 |
| $1.98\times10^1$ | 33.2 | Synthetic | https://doi.org/10.1002/admt.201800419 |
| $2.22\times10^1$ | 47.7 | Synthetic | https://doi.org/10.2140/jomms.2013.8.65 |
| $2.22\times10^1$ | 46.1 | Synthetic | https://doi.org/10.1177/0731684419868018 |
| $2.25\times10^1$ | 47.5 | Synthetic | https://doi.org/10.2140/jomms.2013.8.65 |
| $2.29\times10^1$ | 53.5 | Natural | https://doi.org/10.1016/S0167-6636(02)00268-5 |
| $2.69\times10^1$ | 45.8 | Synthetic | https://doi.org/10.2140/jomms.2013.8.65 |
| $2.86\times10^1$ | 32.1 | Synthetic | https://doi.org/10.1177/0021955X06063519 |
| $3.88\times10^1$ | 45.8 | Natural | https://doi.org/10.1016/S0167-6636(02)00268-5 |
| $5.84\times10^1$ | 34.3 | Synthetic | https://doi.org/10.1016/j.ijsolstr.2015.02.020 |
| $8.86\times10^1$ | 53.6 | Synthetic | https://doi.org/10.1016/j.msea.2004.03.051 |
| $1.40\times10^2$ | 51.5 | Synthetic | https://doi.org/10.1016/j.msea.2004.03.051 |

**Table S1 | Common and superlative structures and materials**

## Supplementary Note 2. Process for Converting Force-Displacement into Stress-Strain

When converting from force-displacement curves to engineering stress-engineering strain curves (simply called stress and strain hereafter), it is necessary to define the area of the component and its height. For traditional materials, this process is straightforward as it amounts to defining the cross-sectional area of the component under study. However, for more complex structures, the area of the component is less clear. Here, we define the cross-sectional area to be the amount of area that is required per component to tile the component infinitely on a plane. To calculate this algorithmically, we used the following steps (illustrated graphically in Figure S2):

1. Find the maximum radius $r_{max}$ of the component by finding the maximum of the radius $r$ at all heights $z$ and azimuthal angles $\phi$, as defined in Equation (1) in the Methods.
2. Enclose the component with a cylinder with radius $r_{max}$ (Figure S2b).
3. Enclose the component in a hexagonal prism that circumscribes the cylinder (Figure S2c).
4. The area of the hexagonal prism is used as an estimate of the cross-sectional area needed to tile the component on a plane.

Although some designs may be more closely packed in a square lattice, most are more closely packed using the hexagonal approach (Figure S2d) due to the applied linear and sinusoidal twists, and this approach is invariant of the rotational orientation of the design. Therefore, this hexagonal packing approach was used to estimate the cross-sectional area of all designs.

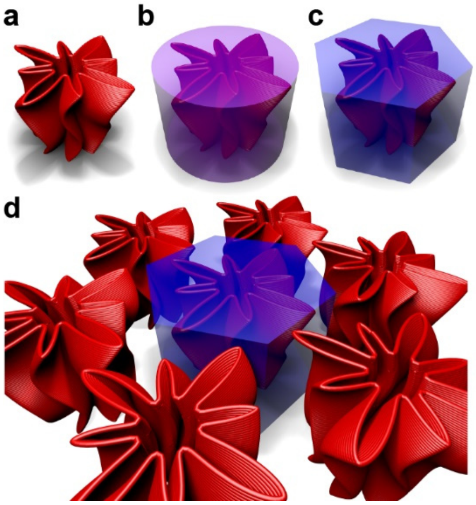


**Fig. S2 | Calculating the effective area of GCS designs. a-b,** To calculate the effective cross-sectional area of a design, it is fit into a cylinder based on its maximum radius. **c,** This cylinder is then enclosed in a hexagonal prism. **d,** The hexagonal prism can be tiled infinitely in a plane. Thus, the effective cross-sectional area of a design is estimated as the area necessary per design to tile it in a plane without collisions.

### Supplementary Note 3. Defining a Generalized Cylindrical Shell

A cylindrical shell is often defined in terms of its height $h$, wall thickness $t$, and diameter $d$. Here, we design generalized cylindrical shells (GSC) that are topologically consistent with cylindrical shells and have a consistent wall thickness and height, but vary in their cross-sectional profile along the axial direction. As a diameter is not an appropriate measure for such a complex shape, we parameterize these using their average perimeter $P_0$. A GCS design is realized by deforming cylindrical shells using three distinct transformations: variable perimeter, variable cross section, and twist (Figure S3a). These transformations are defined mathematically in the methods section of the main text. Briefly, the variable perimeter is realized by linearly varying the perimeter from the top of the GCS to the bottom of the GCS (Figure S3b). In this campaign, the perimeter of the top was constrained to be larger than the perimeter of the bottom to ease with component removal. The cross sections of the GCS were transformed using a summed cosine function (Figure S3c). The top cross section and bottom cross section are specified, and each intermediate layer is calculated as a linear interpolation of these two faces, ensuring a manifold surface. Finally, both sinusoidal and linear twist can be applied to these cross sections in a height-dependent manner (Figure S3d). Collectively, these transforms allow for more than trillions of unique designs.

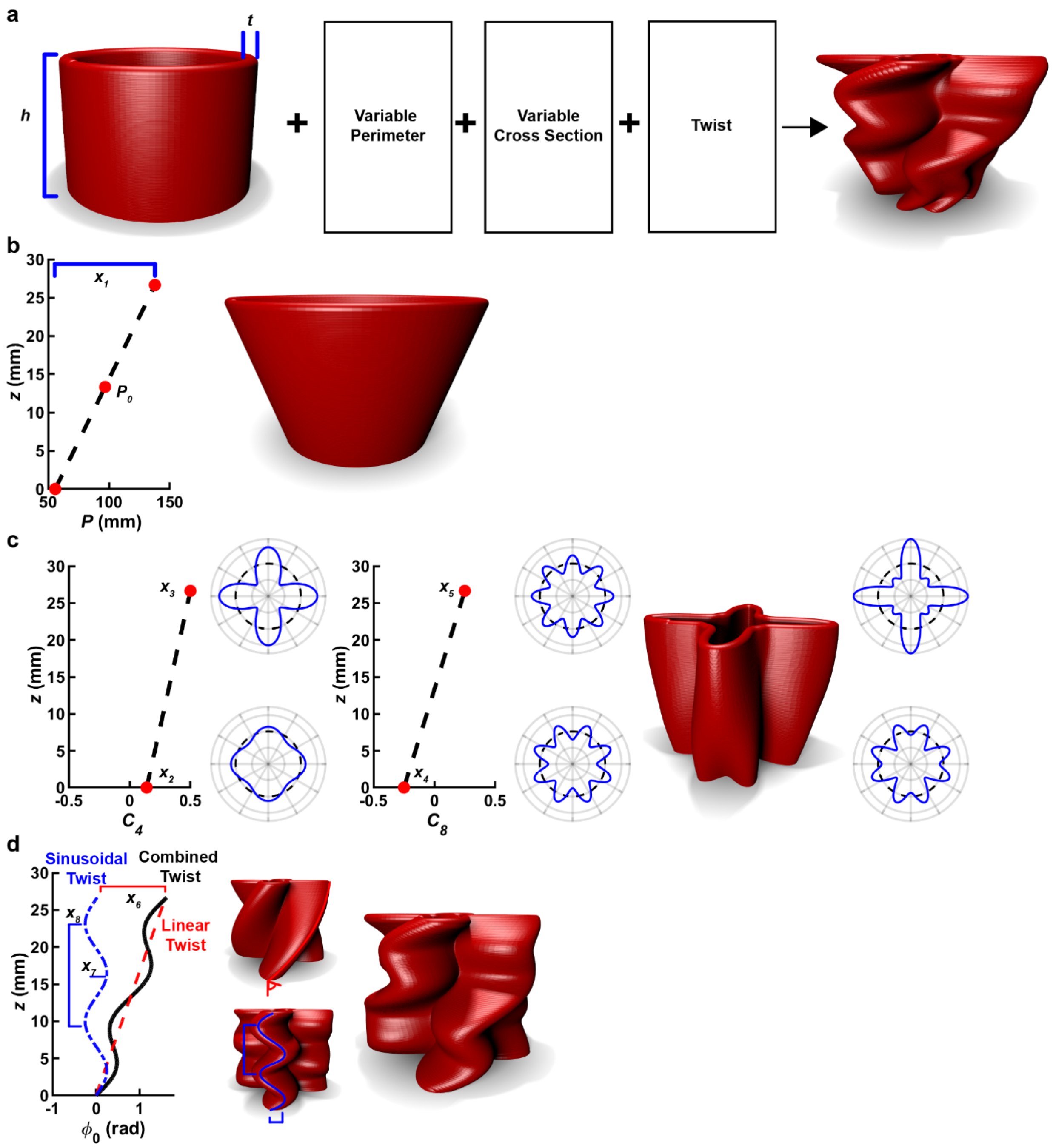


**Fig. S3 | Generalized cylindrical shells. a,** Generalized cylindrical shells (GCS) are realized by transforming a cylindrical shell of height $h$ and wall thickness $t$ to create interesting shapes that preserve the topology of the shell. **b,** The perimeter $P$ varies linearly along the height $z$ of the shell based on an average perimeter $P_0$ and a perimeter difference $x_1$. **c,** The cross sections of each layer are deformed in a $z$-dependent manner using a summed cosine function with 4-period amplitude $C_4$ and 8-period amplitude $C_8$. These are defined at the top and bottom by four variables $x_2$, $x_3$, $x_4$, and $x_5$, and linearly interpolated to determine the cross section at any $z$. **d,** The cross sections of the design are rotated about the cylinder axis in a $z$-dependent manner by rotation angle $\phi_0$ using both linear and sinusoidal twists as defined by linear twist $x_6$, sinusoidal twist amplitude $x_7$, and sinusoidal twist period $x_8$.

## Supplementary Note 4. Polymers under Consideration

The polymer materials studied in this work are provided in Table S2 along with the temperature at which they were printed, the temperature at which the print bed was held during removal, and the material class. In addition, for each spool of material studied, a cylindrical sample was printed and tested to estimate the material properties of the polymer. The details of this process are provided in the methods. As shown in Figure S4, the result of this testing are estimates of the elastic modulus $E$ and plateau stress $\sigma_p$ of each material. In addition, the degree to which the cylinder rebounded after a one-minute relaxation period was also recorded, although this is an imprecise measure of elasticity as a consistent force threshold was used for all tests, indicating that different materials experienced different total strains. Nevertheless, the plastic materials rebounded less than the hyperelastic materials, despite their total strain being lower.

| Material | Manufacturer | Nozzle Temperature (°C) | Bed Removal Temperature (°C) | Class | Spools Used |
|---|---|---|---|---|---|
| TPE (Chinchilla) | NinjaTek | 250 | 100 | Hyperelastic | 9 |
| TPU-1 (NinjaFlex) | NinjaTek | 250 | 100 | Hyperelastic | 16 |
| TPU-2 (Cheetah) | NinjaTek | 250 | 100 | Hyperelastic | 37 |
| TPU-3 (Armadillo) | NinjaTek | 250 | 30 | Intermediate | 11 |
| Nylon | MatterHackers | 250 | 30 | Plastic | 2 |
| PETG | MatterHackers | 250 | 30 | Plastic | 5 |
| PLA | eSun/MakerGear | 220 | 30 | Plastic | 29 |

**Table. S2 | Filaments studied in this work along with their processing settings.**

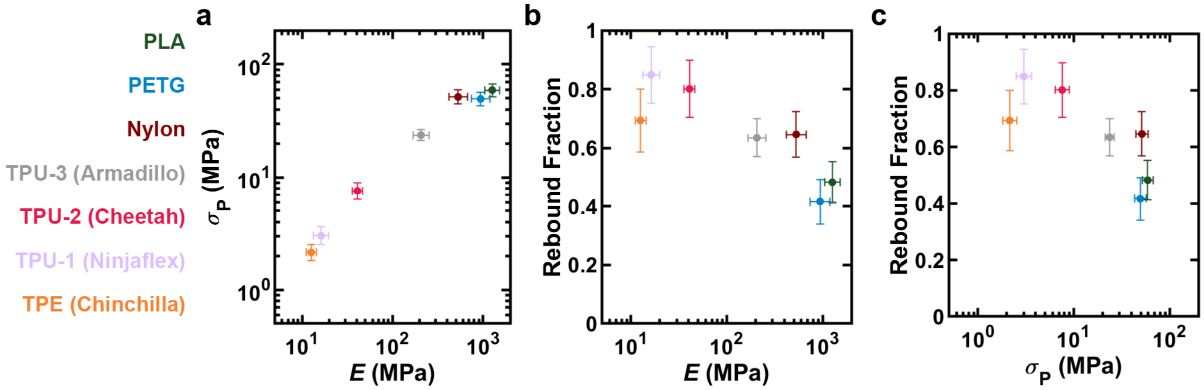


**Fig. S4 | Material characterization of polymers studied. a,** Plateau stress $\sigma_p$ vs. elastic modulus $E$ for seven materials used in this campaign. **b,** Rebound fraction vs. $E$. **c,** Rebound fraction vs. $\sigma_p$. Error bars represent one standard deviation. Here, $\sigma_p$ is the stress at 25% strain. Rebound fraction is the height after 1 minute relaxation divided by the initial height.

## Supplementary Note 5. The Bayesian Experimental Autonomous Researcher

The Bayesian experimental autonomous researcher (BEAR) consists of a collection of computers and other hardware that work together to perform research experiments without direct human intervention. It consists of five fused filament fabrication 3D printers, a scale, a universal testing machine, and a six-axis arm to transfer experiments between the different stations (Figure S5). The various components are controlled centrally by a custom-made MATLAB script (Figure S7). The BEAR has a series of tasks that it can do, which it does in order of a user-modifiable priority (Figure S8).

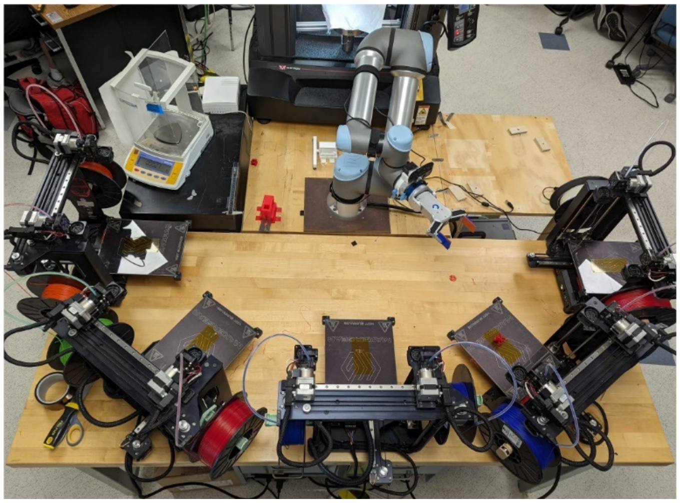

**Fig. S5 | Picture of the Bayesian experimental autonomous researcher (BEAR), consisting of five fused filament fabrication 3D printers, a six-axis robot arm, a scale, and a universal testing machine.**

### 5.1 Select Experiment

Bayesian optimization was used by the BEAR to algorithmically select additional experiments. This process includes the conditioning of a surrogate model to approximate the connection between input space and output space and then the use of an acquisition function to evaluate this model to find experiments that are believed to be most useful to perform. Since the goal of this work was to identify structures with high $K_s^*$, we treated this as a maximization problem. The input space for this maximization was both the design of the GCS and the material used to realize a component out of this design. As such, we required a 13-dimensional input (11 geometric parameters and two material properties). For the output space, we were not just interested in $K_s^*$, but we also found it necessary to predict $\sigma_t^*$. Gaussian process regressions (GPRs) were used to predict $K_s^*$ and $\sigma_t^*$. A neural network with one hidden layer equal to the input size was used to predict component printability. Specifically, predicting printability is done purely through empirical means based on the results of past experiments. Specifically, each experiment is assigned a tag of “printable” or “unprintable” based on a combination of automated processes (*i.e.* was a component delivered to the testing frame) and out-of-the-loop manual inspection. These tags were used to train a classifying neural network that can rapidly predict the printability of any

point in the GCS parameter space. To evaluate potential experiments, the output of this neural network was multiplied by their predicted $K_s^*$ to bias the search into printable regions of parameter space.

In developing surrogate models, transformations were done to the GCS design parameters and material parameters to improve the accuracy of the models. The overall motivation of these transformations was to improve correlations in the input space and thus improve predictions in the output space. The full list of the transformed input and output spaces are given in Tables S3 and S4. For example, the logarithms of $\sigma_p$, $\sigma_t^*$, and $E$ were taken because their values varied over several orders of magnitude and the points were more evenly spaced when considered logarithmically rather than linearly. Additionally, rather than specify $P_0$, we preferred to specify the target mass $m$ normalized by $h$, or the mass per height $m/h$. Additionally, a wall angle $\theta$ was used as it was hypothesized that the angle of the wall was more important than the absolute value of the change in $P$. This wall angle was estimated using the formula $\theta = \text{atan}\left(\frac{x_1}{2\pi h}\right)$. Finally, rather than conditioning the GPR to directly predict $K_s^*$, we found that it was useful to transform $K_s^*$ to emphasize differences at the high end while minimizing differences at the low end and to explicitly prevent the model from predicting physically impossible values (*i.e.* $K_s^*>1$ or $K_s^*<0$). Thus, we instead predicted $\text{atanh}(2K_s^*-1)$. This function was chosen because it monotonically transforms inputs from 0-1 to outputs from negative infinity to positive infinity. Mechanical intuition suggests that a given design produced using different materials may have a similar efficiency, but its strength should vary based upon the strength of the material. Given sufficient time, this trend would naturally be learned by an active learning process. However, to accelerate this process, we hypothesized that learning the strength of a design relative to its material properties may be more efficient. Indeed, early in the campaign we plotted $\log \sigma_t^*$ vs. $\log E$ and found that the best fit trend line had a slope of 0.408. Thus, we began normalizing $\sigma_t^*$ by $E^{0.408}$ in an attempt to capture this variation of component strength with material properties. It is important to note that variations about this trend were still allowed for and learned.

| Model input variable | Description |
|---|---|
| $h$ | Height (mm) |
| $m/h$ | Mass per height (g/mm) |
| $t$ | Wall thickness (mm) |
| $\text{atan}\left(\frac{x_1}{2\pi h}\right)$ | Wall angle (degrees) |
| $x_2$ | 4-period amplitude of bottom cross section (dimensionless) |
| $x_3$ | 4-period amplitude of top cross section (dimensionless) |
| $x_4$ | 8-period amplitude of bottom cross section (dimensionless) |
| $x_5$ | 8-period amplitude of top cross section (dimensionless) |
| $x_6/h$ | Linear rotation per height (rad/mm) |
| $x_7$ | Sinusoidal rotation amplitude (rad) |
| $x_8$ | Sinusoidal rotation wavelength (mm) |
| $\ln(E)$ | Natural log of the polymer elastic modulus ln(MPa) |
| $\ln(\sigma_p)$ | Natural log of the polymer plateau stress ln(MPa) |

**Table. S3 | Inputs to the machine learning models used for Bayesian optimization.**

| Model output variable | Description | Model type |
|---|---|---|
| atanh($2K_s^*-1$) | Transformed peak energy absorbing efficiency (dimensionless) | Gaussian process regression |
| $\log\left(\frac{\sigma_t^*}{E^{0.408}}\right)$ | Log 10 of the ideal threshold stress normalized by the modulus and raised to an empirically determined power log($MPa^{0.592}$) | Gaussian process regression |
| $p$ | Printability (dimensionless) | Artificial neural network |

**Table. S4 | Outputs of the machine learning models used for most of the experimental campaign.**

When selecting a subsequent experiment for a given printer, not all combinations of designs and materials were available. Specifically, each printer had two independent extruders, which allowed two different filaments to be loaded at once. Further, each extruder had either a 0.5 mm diameter nozzle or a 0.75 mm diameter nozzle. Values of $t$ different from these diameters could be achieved by over or under extruding. We restricted $t < 0.7$ mm for the 0.5 mm diameter nozzle and $t \geq 0.7$ mm for the 0.75 mm nozzle.

To select an experiment, we define an acquisition function $a$ that takes as its input positions in parameter space along with the current surrogate models and select the experiment that maximizes $a$. Throughout the campaign, three types of acquisition functions were used: maximum variance ($a$ is equal to the variance in predicting $K_s^*$), expected improvement ($a$ is the predicted amount of improvement beyond the previous best $K_s^*$), and upper confidence bound ($a$ is the weighted sum of the predicted of $K_s^*$ and the predicted uncertainty in predicting $K_s^*$). The combination of $a$ and the strategy for finding its maximum is considered a decision policy. However, this process was not treated as a simple single-objective maximization. For instance, in all cases, $a$ is multiplied by the predicted printability $p$ to ensure that we are only considering components that are expected to be realizable in practice. Additionally, many of the decision policies are multi-objective, trying to find high values of $K_s^*$ across a range in $\sigma_t^*$. When this was the case, multiple GPR model predictions were combined to select a component by penalizing the $K_s^*$ prediction by the distance of its predicted $\sigma_t^*$ from the target $\sigma_t$ or by comparing the predicted $K_s^*$ to the performance of other tests at that $\sigma_t^*$. A full list of considered decision policies is given in Table S5. These policies were added sequentially during the progression of the campaign, so their order reflects the evolution of our thought process during the campaign, discussed further in Section 6. Additionally, the GPR models can be retrained using only data from the region of interest, which was begun with decision policy 19. This allowed the GPR to capture finer correlations in the parameter space around the region of interest. All models were trained using MATLAB's built in functions and the code is available at https://github.com/KelseyEng/BEAR_ADTS. Model Training was performed on Boston University's Shared Computing Cluster, where multiple compute nodes could work in parallel. GPR processing time scales with the number of experiments cubed.[1] Therefore, the longer the campaign ran, the more computationally expensive model building and component selection became.

| **Decision Policy Number** | **Acquisition function** | **Metric** | **Number of Valid Experiments** |
|---|---|---|---|
| 0 | Manually Selected | Researcher intuition or performance validation | 730 |
| 1 | Upper confidence bound | Full integral of force-displacement curve | 24 |
| 2 | Maximum variance | Full integral of force-displacement curve normalized by component mass | 916 |
| 3 | Expected improvement | Full integral of force-displacement curve normalized by component mass | 775 |
| 4 | Expected improvement | Expected acceleration of a simulated impact test | 93 |
| 5 | Expected improvement | $K_s$ at a target $\sigma_t$ | 249 |
| 6 | Expected improvement | $K_s^*$ penalized by an amount proportional to the distance between $\sigma_t^*$ and a target $\sigma_t$ | 97 |
| 7 | Expected improvement | $K_s^*$ penalized by an amount proportional to the distance between $\sigma_t^*$ and a target $\sigma_t$ with uncertainty in $\sigma_t^*$ considered | 383 |
| 8 | Expected improvement | $K_s^*$ minus the best $K_s$ previously observed at the predicted $\sigma_t^*$ | 3,219 |
| 9 | Expected improvement | $K_s^*$ minus the best $K_s$ previously observed at the predicted $\sigma_t^*$, but with limits imposed on the largest and smallest stresses considered | 31 |
| 10 | Expected improvement | $K_s^*$ minus the best $K_s$ previously observed at the predicted $\sigma_t^*$, but only considered components that could have been printed using the specific printer under consideration | 501 |
| 11 | Maximum variance | $K_s^*$, but only considering cylindrical shells | 34 |
| 12 | Expected improvement | $K_s^*$ | 1,608 |
| 13 | Not Used | | |
| 14 | Expected improvement | A weighted sum of the acceleration from a simulated impact test and the plateau stress of the component | 41 |
| 15 | Expected improvement | A weighted sum of the acceleration from a simulated impact test and the plateau stress of the component (different simulation model from DP 14) | 212 |
| 16 | Expected improvement | $K_s^*$ times the ideal threshold force for that component | 22 |
| 17 | Expected improvement | $K_s^*$ minus the best $K_s^*$ that could have been printed using the specific printer under consideration | 1,041 |
| 18 | Expected improvement | $K_s^*$, but only considering components near the best previously found component | 1,569 |
| 19 | Upper confidence bound | $K_s^*$, but only considering components near the best previously found component | 224 |
| 20 | Upper confidence bound | $K_s^*$, but only considering components near the best previously found component that have effective densities $\rho_d$ below 10% | 542 |
| 21 | Not Used | | |
| 22 | Expected improvement | $K_s^*$ minus the best $K_s$ previously observed at the predicted $\sigma_t^*$, but only considering components that could be continuously extruded without a linear twist | 523 |
| 23 | Expected improvement | $K_s^*$ minus the best $K_s$ previously observed at the predicted $\sigma_t^*$, but only considering components that could be continuously extruded with a linear twist | 286 |

| | | | |
|---|---|---|---|
| 24 | Expected improvement | $K_s^*$ of a two-component system | 129 |
| 25 | Expected improvement | $K_s^*$ of a two-component system, but only considering components near the best previously found pair of components | 1 |

**Table. S5 | Descriptions of decision policies used during campaign.**

Initially, sampling points were selected on a grid. Starting at ID 9,261, potential sampling points were selected using Latin hypercube sampling (LHS) to facilitate exploring space more finely. Starting with ID 11,763, after the proposed experiment had been selected, a second round of sampling points were added that were zoomed in a hypercube around the selected point to more closely find the maximum of $a$.

## 5.2. Generate G-code

Once a component has been selected for testing, the STL was generated using a custom Python script. This Python script (Python version 3.8.3) was run on the main computer and called from MATLAB using the command line function. The resulting STL was created as a solid object. In order to convert this STL file into the G-code needed for the printer, Slic3r (version 1.3.0) was run from the command line of MATLAB. Prior to sending the STL file, the Slic3er configuration file was edited using string manipulation directly from MATLAB to set the nozzle temperature, bed removal temperature, and extrusion multiplier. The two temperatures were designated by the human team based on our experience with these materials (see Table S2) while the extrusion multiplier was set as part of a feedback system to maintain component weight (see below). Slic3er was configured to use vase mode (spiral mode), which removes the tops and bottoms of solid objects and turns the STL solid into a shell. The output of this process is G-code for the print and predicted amount of filament that is needed to print this component, which is read into MATLAB. Using an initial set of calibration prints and subsequent use of integral feedback, we predicted the mass of the component from the amount of filament predicted to be used by the slicer. Adjusting the extrusion multiplier and reslicing the component provided a reliable method of controlling the mass of the final component and standardizing performance across different printers and filament rolls (Figure S6). This gives effective control over $t$ by over or under expanding the material leaving the nozzle. It also allows the computer to automatically compensate for variations in the thickness of the filament diameter or variations between the stepper motor of different printers. Relatively slow print speeds of 15 mm/min were employed to prevent clogging, which was especially important for the softer filaments.

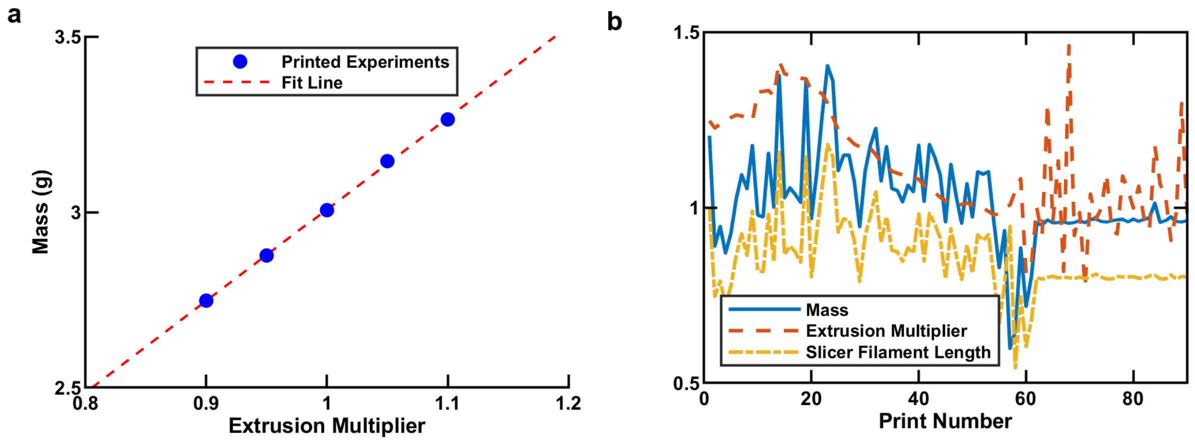


**Fig. S6 | Mass calibration through extrusion multiplier adjustments. a,** A single component printed five times with different extrusion multiplier shows that component mass is linearly correlated with extrusion multiplier. **b,** Applying integral tuning to the extrusion multiplier was ineffective because of variations in slicing complex curved structures, as seen in print number < 60. However, when integral tuning was applied to the slicer filament length by adjusting the extrusion multiplier rapidly, consistent mass was obtained (print number > 60). Mass is normalized by target mass and slicer filament length is normalized by the initial slicer filament length (print number = 1).

## 5.3 Begin Experiment

Once the G-code had been prepared for a given printer, the physical experiment was ready to begin. First, the arm moved into position over the chosen printer and the arm-mounted camera took a photograph of the print bed to ensure that it was free from debris and ready for the next print. To accomplish this, the picture was run through a neural net based on GoogLeNet[2] and classified as 'clear' or 'needs cleaning'. If the bed needed cleaning, the robot arm picked up a scraper and scraped the print bed. A new picture was then taken to verify that the print bed was clean. If the bed was still unclean, the BEAR would attempt to clean the bed up to ten times with the scraper. If the bed was still unclean, the printer would be deactivated and the operator was notified to clean the bed before further experiments. Once the bed was determined to be clear, the system sent G-code to the printer using OctoPrint.

## 5.4 Weigh Component

When a print was complete, as determined by querying the state of the printer through OctoPrint, the bed was heated (TPE, TPU-1-2) or allowed to cool (PLA, PETG, and Nylon, TPU-3) to facilitate removal of the component.[3] Once the desired temperature had been reached, the robot arm removed the component from the printer and moved it to the scale, which determined its mass. This mass reading was read through a serial port by MATLAB. If no mass was registered on the scale, the system attempted to re-grab the component from the print bed up to three times. At this stage, the arm-mounted camera took a photograph of the component on the scale, which

was used to verify that the component was fully on the scale. Components that were misoriented, as determined by machine vision, were discarded before testing.

### 5.5 Test Component

If a component was on the scale and ready to be tested while the universal testing machine (UTM) was not performing any experiments, the component was moved to the UTM for compression testing. Once the component was in position, the main computer sent a command to the Instron computer to begin the test through a .mat file transferred by the cloud. The Video computer then told the Instron to start the test while it recorded a video of the compression testing. The test began with the top platen ~200 mm over the component. After zeroing the force sensor, the top platen moved at a rate of 50 mm/min toward the component until the force sensor registered 1 N. The platen then moved away from the component 1 mm so that it no longer was in contact with the component. At this point, the UTM started recording the force measurement while it lowered the top platen at 2 mm/min. A given test ended when either 1) the force exceeded the 4.5 kN force limit or 2) the top platen position fell below the safe height of 0.4 mm separation between the two platens. After a one minute relaxation period, the platen was lowered again at a rate of 100 mm/min until the force exceeded 1 N to find the rebound height. After testing, the component was removed from the UTM and stored. The platen was then cleaned with the robot arm to ensure that the platens were clear and ready for the next test. Each mechanical test took approximately ten minutes. A third computer recorded the Instron data and saved it to the cloud. When the test was finished, the Video computer informed the main computer that the UTM was now free for another experiment.

### 5.6 Process Results

When new experimental results were available to be processed, the raw force-displacement data was loaded into MATLAB. The as-printed height of the component was calculated by finding the platen separation when the moving median of twenty force measurements surpassed 0.3 N. The effective area of the component was calculated by computing the maximum radius of any layer of the component and using that as the apothem (distance from center to midpoint of side) of a circumscribing hexagon (Figure S2). Using this height and effective area, the force-displacement curve was converted to a stress $\sigma$ – strain $\varepsilon$ curve.

From the $\sigma$–$\varepsilon$ curve, a variety of useful metrics were calculated. To find $K_s^*$ and $\sigma_t^*$, $K_s$ was calculated at 1,400 $\sigma_t$ values that were logarithmically spaced between 10 Pa and 100 MPa. Ten additional $\sigma_t$ sampling points were selected by diving the $\sigma$–$\varepsilon$ curve into ten equal sections in $\varepsilon$ and finding the maximum $\sigma$ in each section. Because $\sigma_t^*$ is often a peak early in the $\sigma$–$\varepsilon$ curve, these ten additional sampling points can often determine $\sigma_t^*$ precisely. For each of these sampling points (1,400 evenly spaced points and ten extra points taken from the $\sigma$–$\varepsilon$ curve), we compute $K_s(\sigma_t)$ and assign $K_s^*$ and $\sigma_t^*$ to the maximum and argmax of this calculation, respectively. The densification strain $\varepsilon_d$ is the $\varepsilon$ value at which $\sigma$ first exceeds $\sigma_t^*$. The relative density of the component $\rho_r$ was

calculated by dividing the mass of the component by the mass of solid material equal to the volume of the enclosing hexagon (Figure S2).

Finally, quality control checks were performed to determine if the sample should be included in the complete dataset. Components that were not within 5% of their mass target or within 5% of their target height were excluded from the results. Additionally, components that hit the force threshold of the UTM when $\varepsilon < 0.3$ were excluded due to the high probability that $\sigma_t^*$ was greater than the UTM's force threshold.

### 5.7 Maintenance

At the beginning of the campaign and periodically thereafter, new filament rolls were loaded into the printers. After performing material characterization (Section 3), a series of calibration components were printed to tune the extrusion multiplier of the printer to the density and diameter of the filament. The target mass for the calibration component was 3.3 g. If the component was too heavy, the extrusion multiplier was decreased. If it was too light, the extrusion multiplier was increased. This continued until the mass was within 5% of the target mass. In this way, it was possible to estimate the ratio of the filament length computed by Sli3er to the mass of the resulting component. As components were subsequently printed during the campaign, this ratio was slowly adjusted using integral tuning to remain accurate. Additionally, components were printed on polyimide tape that was applied to the glass bed of the printers. Whenever the tape showed signs of wear or became damaged, it was manually replaced.

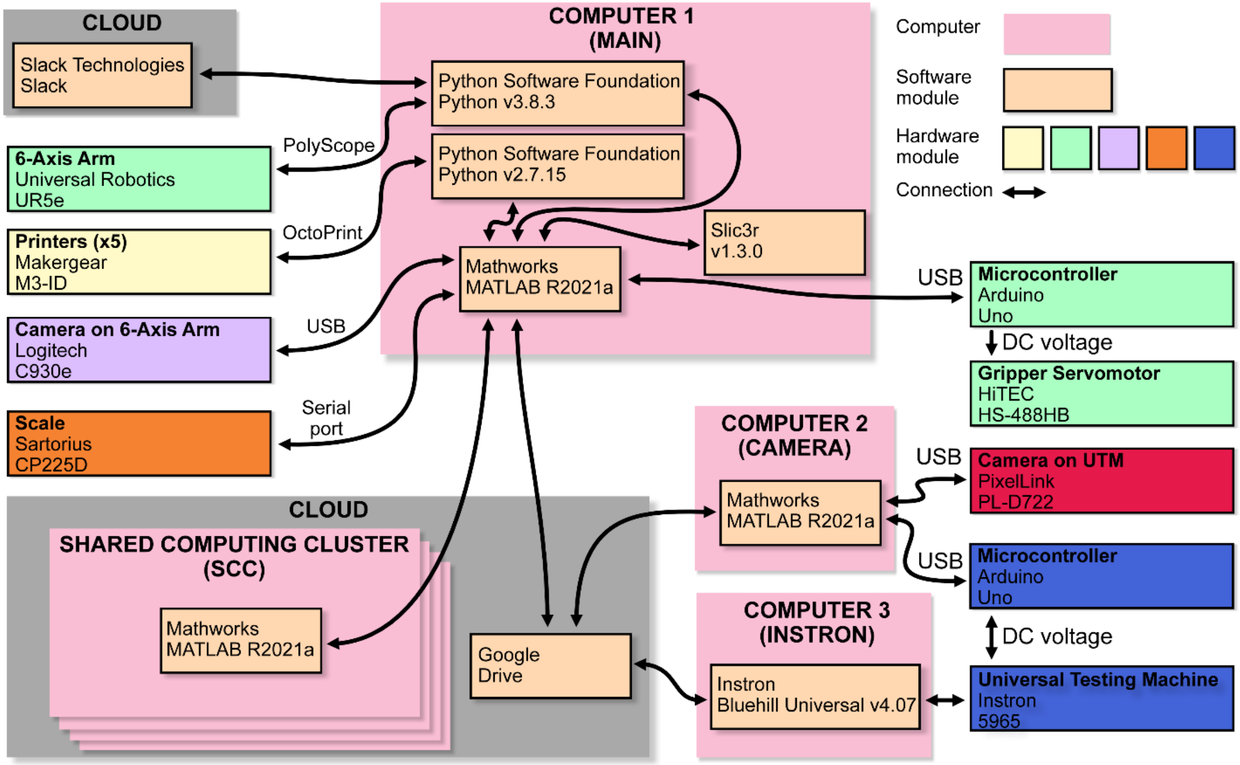


**Fig. S7 | Hardware and software Organization of the Bayesian experimental autonomous researcher (BEAR).**

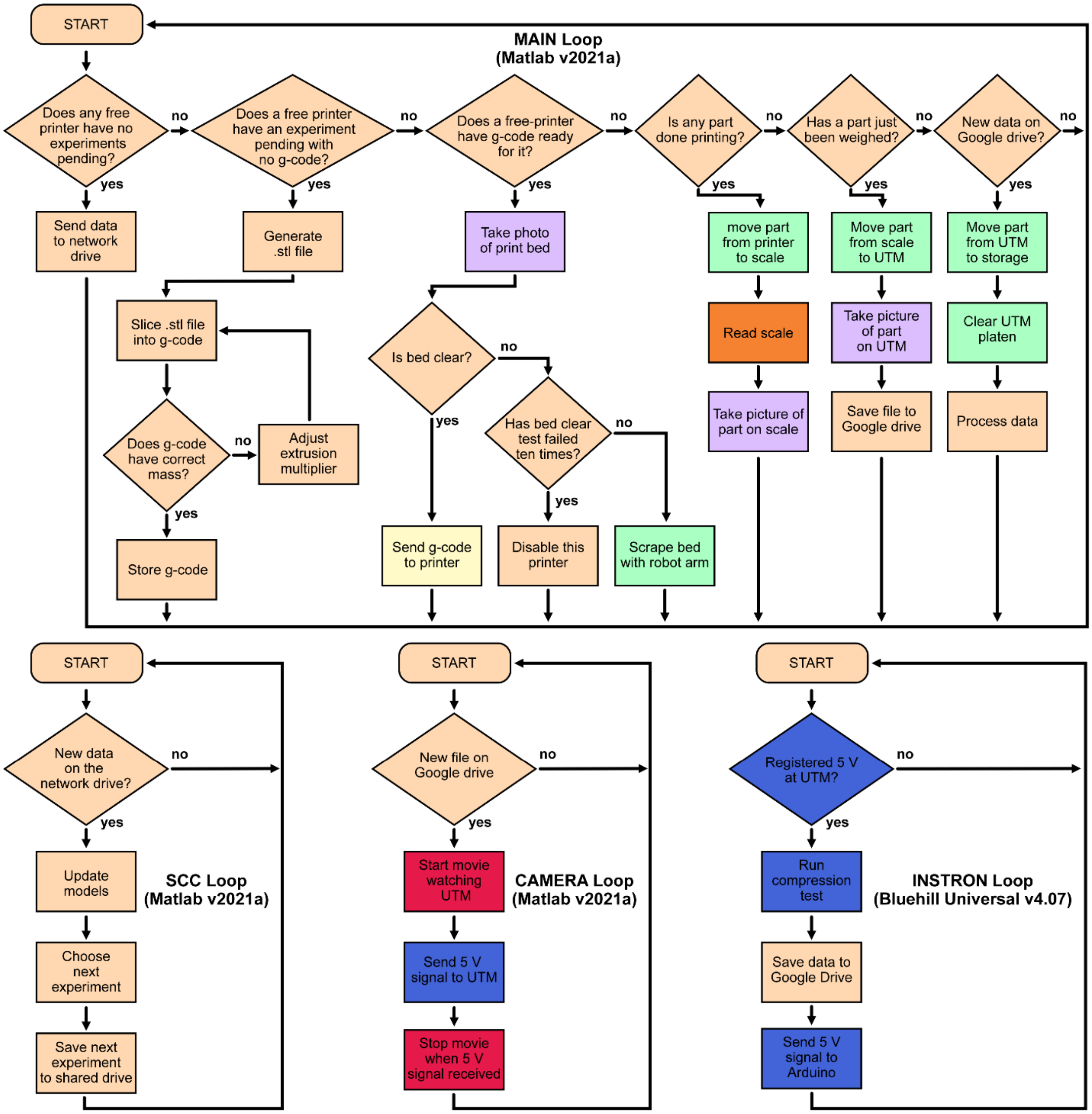


**Fig. S8 | Flowchart of the software loops executed in the four computers running as part of the BEAR.** Colors on the panels correspond to systems in Figure S7. Order of Main Loop actions can be adjusted by researchers to maximize throughput by prioritizing potential bottlenecks.

## Supplementary Note 6. Details of the Experimental Campaign

The experimental campaign consisted of 25,387 experiments. During the course of the campaign, the available search space was changed by adding parameters, changing the limits of the included parameters, and by changing the method used to sample search space. After each experiment, the results were evaluated for defects in fabrication or testing. Components were excluded from the database if their height or mass deviated more than 5% from the target or if the maximum strain recorded was less than 30%. Additionally, researchers excluded components with severe print defects, which were reviewed daily. A record of all experiments performed is provided in Figure S9 and the raw data associated with these experiments is shared via kablab.org/data.

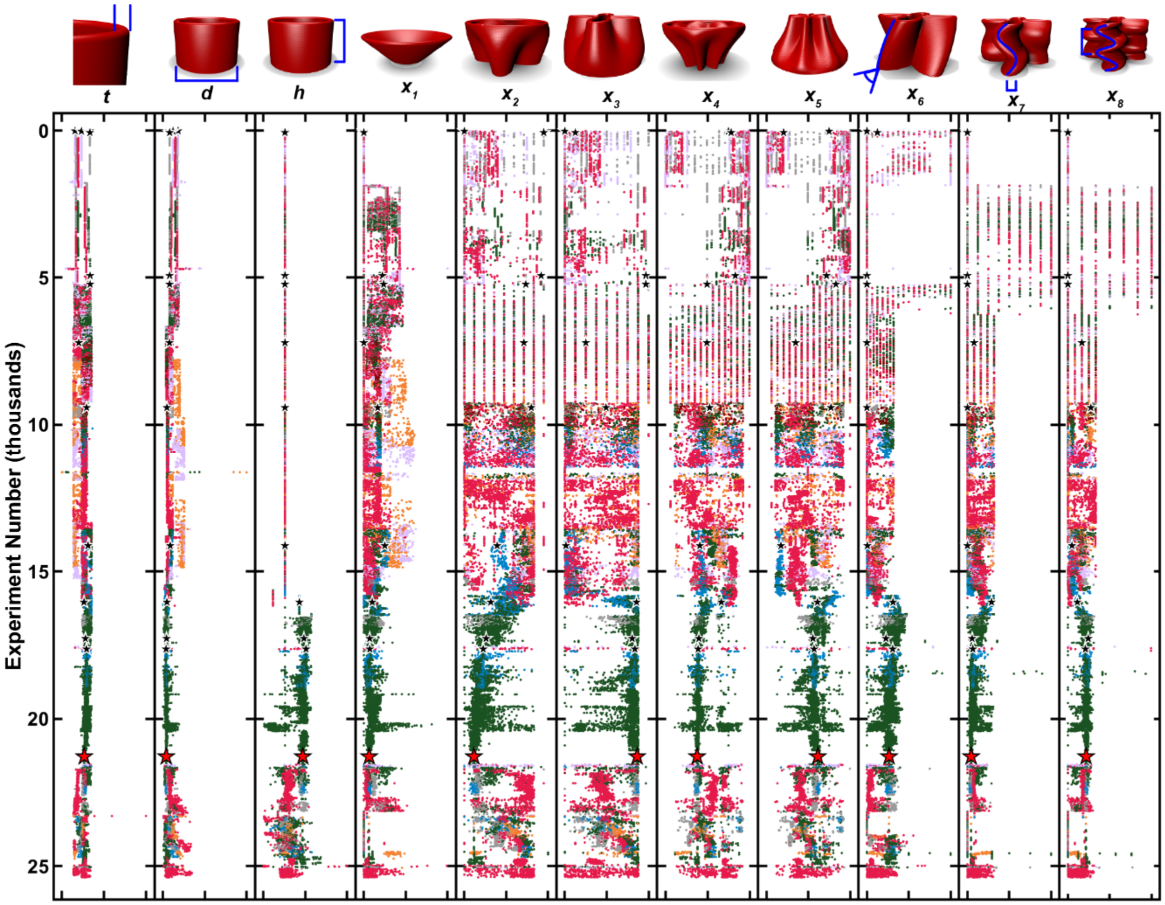


**Fig. S9 | Experiments carried out by the BEAR.** Experiments performed during the campaign, which are defined by eleven GCS parameter values. The color of each dot corresponds to the material used, as designated in Figure S4. Black and red stars correspond to breakthrough experiments, as designated in Figure 2a.

Over the course of the multi-year campaign, the details of how experiments were chosen were altered based on the intuition of the experimenters and by evaluating the progress of the BEAR. Examples of these changes include, the introduction of sinusoidal twist, the switch to LHS sampling (from grid-based sampling), allowing components to have both sinusoidal and linear

twists combined, and switching to cooling plastic materials after printing. The timing of these changes is shown in Figure S10a. Researchers also controlled which filaments were loaded into which nozzles. New filaments were introduced during the campaign and the mix of filaments was changed to pursue different goals, as summarized in Figure S10b. Finally, 23 different decision policies were used throughout the campaign, as shown in Figure S10c and Table S3. Of particular importance was the introduction of $K_s^*$ as a key metric in decision policy six and the introduction of GPRs created by zooming in on the region of the best component to date, introduced with decision policy 18.

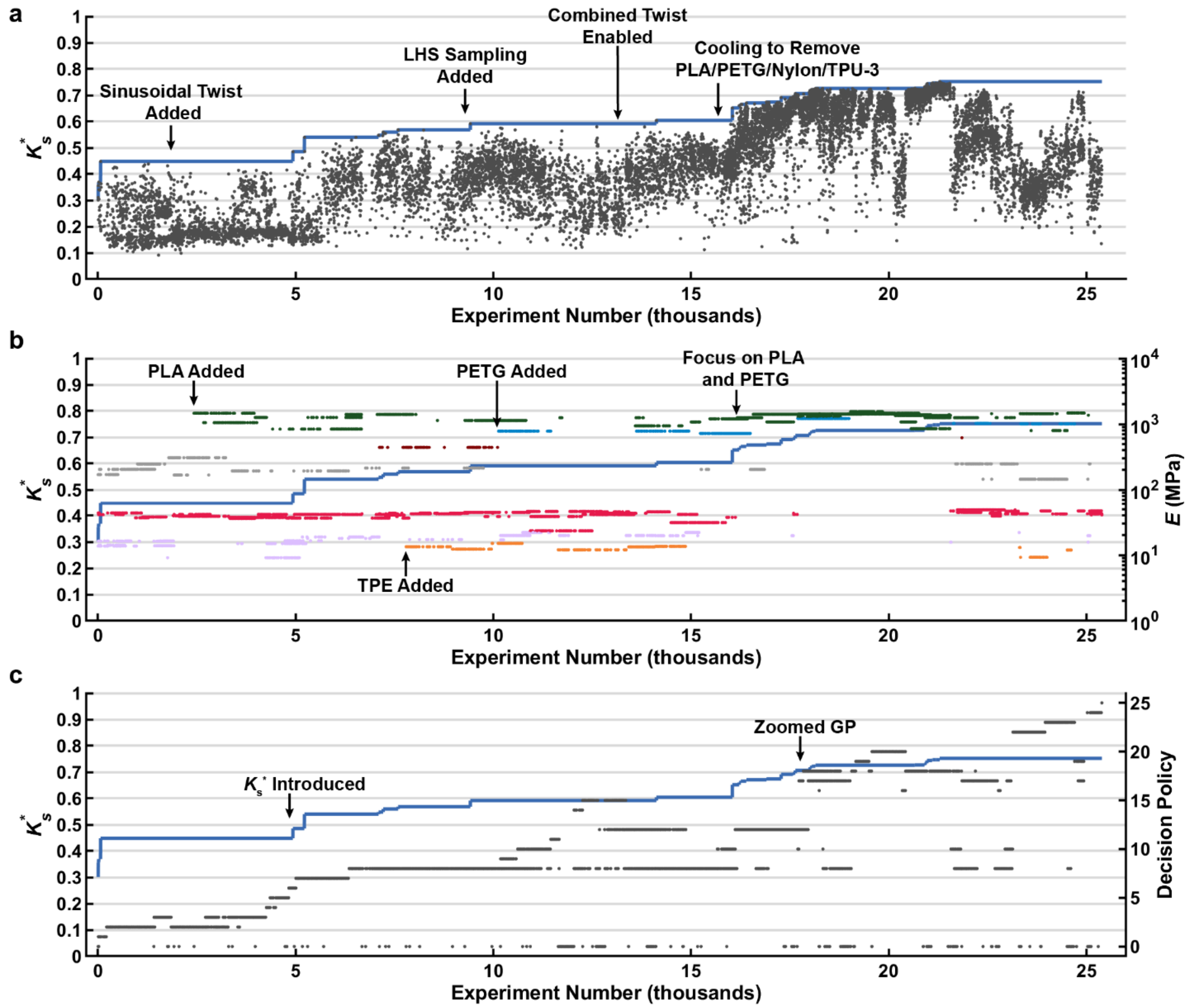


**Fig. S10 | Details of the human/machine collaboration. a**, $K_s^*$ of each successful test (gray dots), along with the highest $K_s^*$ to date (blue line). Key changes to the processing and sampling space are marked. **b**, Modulus of each experiment's filament roll plotted in semi-log (right axis) and colored according to the Figure S4, along with the highest $K_s^*$ to date (blue line – left axis). **c**, Decision policy of each experiment (right axis), along with the highest $K_s^*$ to date (blue line – left axis). Decision policies are listed with descriptions in Table S3.

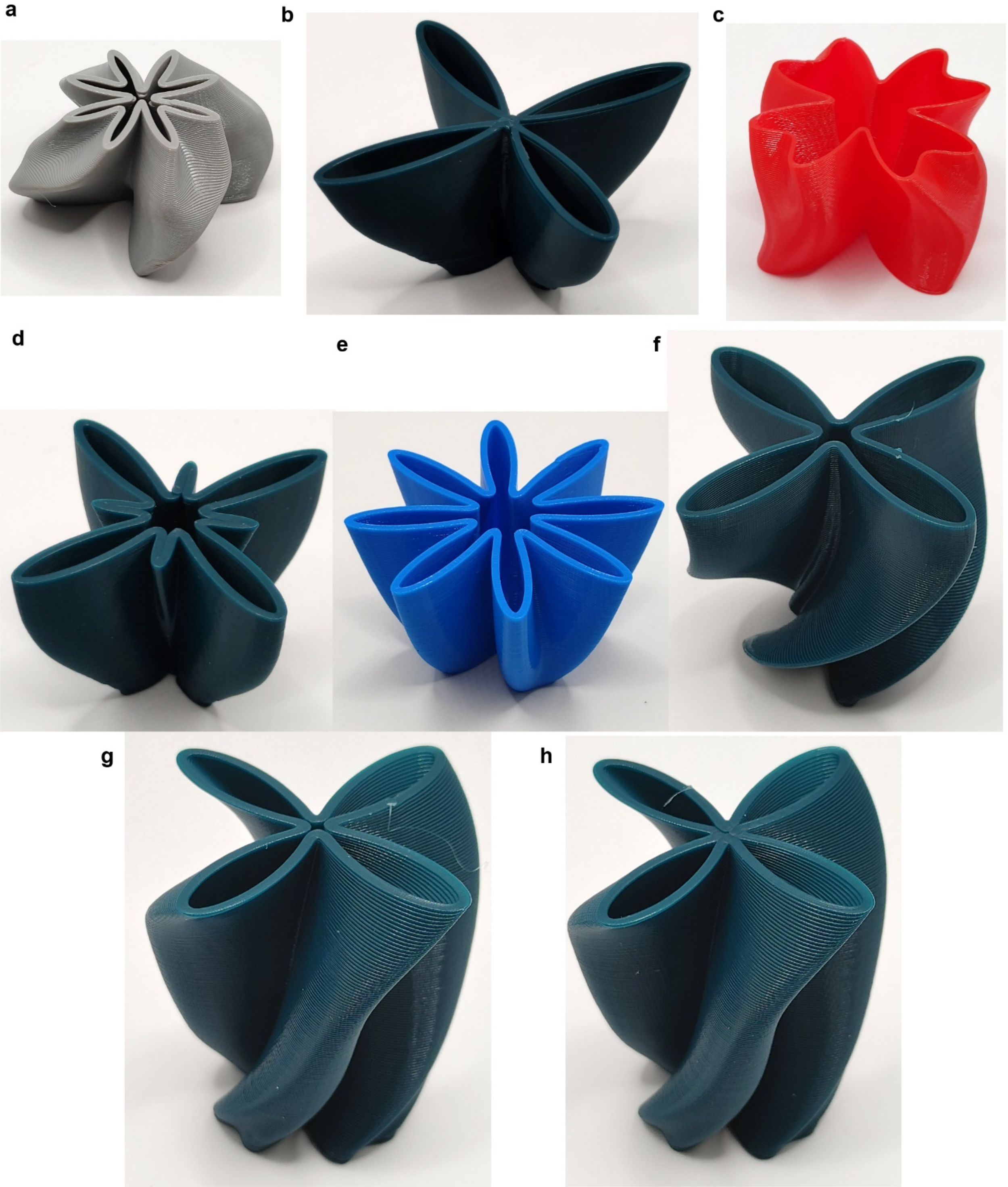


**Fig. S11 | Enlarged images of noteworthy components. a-h**, Pictures of noteworthy parts that significantly improved $K_s^*$ during the course of the campaign, as seen in Figure 2a. Heights vary from 19 mm (a-d) to 27.8 mm (g). Maximum widths range from 29 mm (g) to 48 mm (b). The color of the pictured components is indicative of the material used, with Green indicating PLA, Blue indicating PETG, Red indicating TPU-2, and Gray indicating TPU-3. Pictures are reprints, as the original parts were deformed during initial testing.

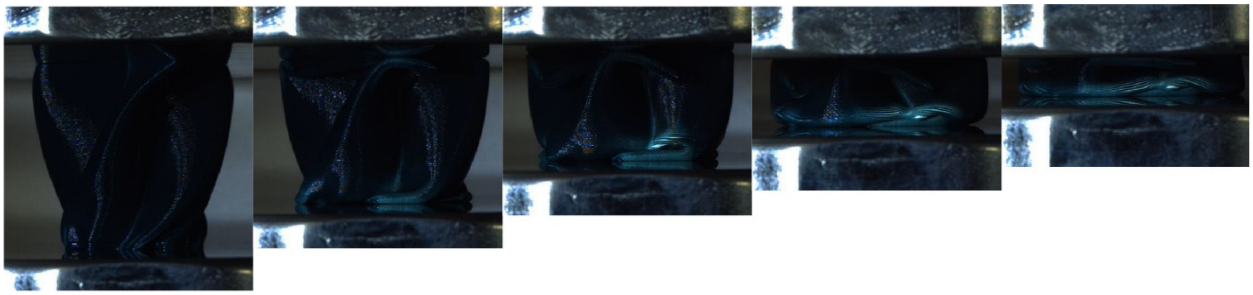

**Fig. S12 | As-recorded and uncorrected images for experiment 21,285.** Still images obtained from the video of this component being tested in uniaxial compression. Enhanced images and their corresponding strain are shown in Figure 2b.

## Supplementary Note 7. Post-Analysis of the Campaign

Following the conclusion of the campaign, we sought to use the corpus of test results to understand the performance of superlative designs (Section 7.1), the performance envelopes of each material (Section 7.2), and the use of game theory to tease out the parameters responsible for the performance of the most efficient components (Section 7.3).

### 7.1 Comparison of the Iroko and Willow Designs

The superlative designs discovered in hyperelastic and plastic materials were very different. Plastic materials, which deform permanently, were able to achieve $K_s^* > 75\%$. Components made from hyperelastic materials, in contrast, were all $K_s^* < 63\%$ with consistent values being significantly lower still. Performance for superlative components made using the same design but different materials was correlated within material classes, but decreased significantly when moving outside the material class. For the top performing PLA design (Willow) and the top performing TPU-2 design (Iroko), three samples were printed on each of the five printers, for a total of 15 samples. Additionally, three samples were printed on a single printer for four other materials. All of these $\sigma$–$\varepsilon$ curves are shown in Figure S13. Of particular interest, several of the TPU-3 and PETG components experienced buckling events during compression, leading to sharp declines in the force that recovered at higher values of displacement (Figure S13b). Because the Iroko design utilizes elastic wall bending to absorb energy, stiffer plastics can buckle abruptly, leading to decreased efficiency. This is an example of how the mechanical outcome can depend on the confluence of material properties and structure.

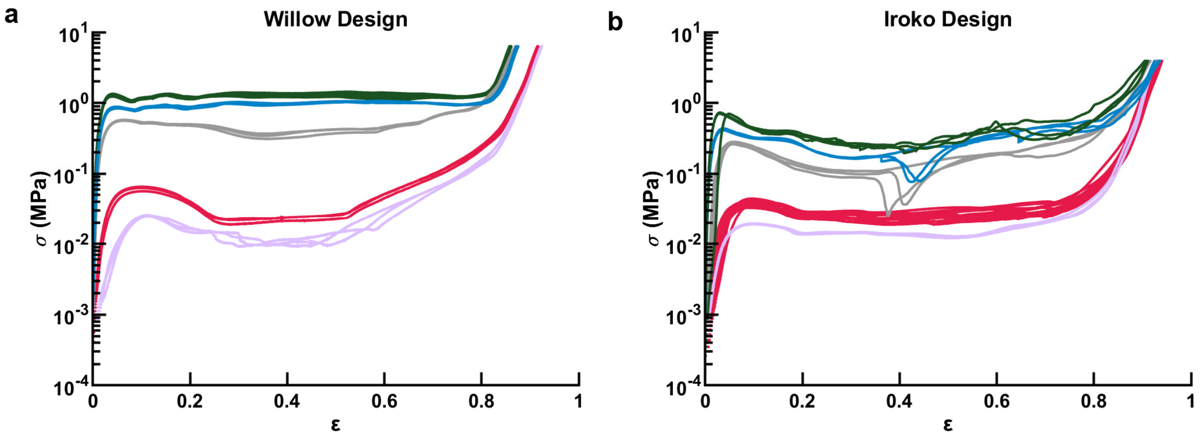


**Fig. S13 | Willow/Iroko by material. a**, Stress $\sigma$–strain $\varepsilon$ curves for components made using the Willow design printed in TPU-1, TPU-2, TPU-3, PETG, and PLA. PLA, the original Willow material, has 15 tests, while the other materials have three each. **b**, $\sigma$–$\varepsilon$ curves for components made using the Iroko design printed in the same five materials. TPU-2, the original Iroko material, has 15 tests while the other materials have three each. Colors depict the material as in Figure S4.

## 7.2 Material-Dependent Performance Envelope

The attainable envelope of $K_s^*$ and $\sigma_t^*$ for each material was estimated by computing a convex hull around all experimentally measured points (Figure S14). To determine the maximum stress $\sigma_{tp}$ for each material, the point with the highest $K_s^*$ was chosen. To obtain a measure of the uncertainty in this term, we retroactively step through the campaign and determine each time the $\sigma_{tp}$ would change and report the expected value as the median of these terms with the error being the standard deviation in their values (in logarithmic space).

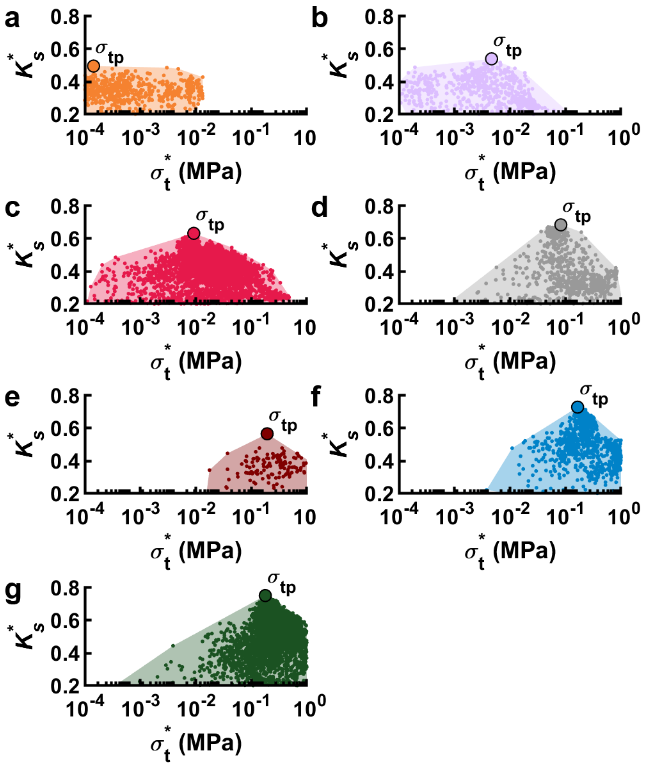


**Fig. S14 | Convex hulls for seven materials.** All tests for each of the seven materials studied, with their final $\sigma_{tp}$ marked. The materials are TPE (a), TPU-1-3 (b-d), nylon (e), PETG (f), and PLA (g).

### 7.3 Statistical Analysis of the Superlative Design

To understand how GCS parameters influence superlative components, we employed a machine learning-based approach to assess the significance of these parameters. Specifically, we built a neural network to predict $K_s^*$ for components made out of PLA. Figure S15a depicts the parity plot of this network. By applying Shapley additive explanations (SHAP),[4] we were able to separate the individual contributions made by each GCS parameter to the neural network's predictions of $K_s^*$. Inspired by Shapley values in Game Theory, SHAP assigns a value to each feature in a machine learning model, indicating its impact on the prediction. We seek to understand the difference in influence between a component and an ideal cylindrical shell (same diameter, height, and wall thickness). To achieve this, we subtract the SHAP values of Willow from the SHAP values for a pure cylindrical shell to obtain a "delta" in explanations. Our analysis of Willow revealed that the four most influential parameters contributing to its predicted performance are the wavelength of the sinusoidal twist ($x_8$), the linear twist linear ($x_6$), and the 4-period amplitude of the bottom and top ($x_2$, $x_3$), (Figure S15b).

The neural network used for SHAP analysis comprised six layers: a 64-dimension linear layer followed by a ReLU activation[5], repeated three times. A data split of 80% for training, 10% for validation, and 10% for testing was employed. The GCS parameters were normalized and no preprocessing was applied to $K_s^*$. The network was trained using the mean squared error loss

function. The training process uses the Adam optimizer[6] with a learning rate of 0.001, weight decay of $1\times10^{-5}$, and a batch size of 16. Training was performed for 500 epochs with early stopping. The PLA network achieved a test loss of 0.0032 and a coefficient of determination $R^2$ = 0.88. For interpreting the predictions generated by the neural networks, we used the SHAP DeepExplainer which is initialized using the training split data. To provide explanations for individual components, we use the default SHAP waterfall visualization.

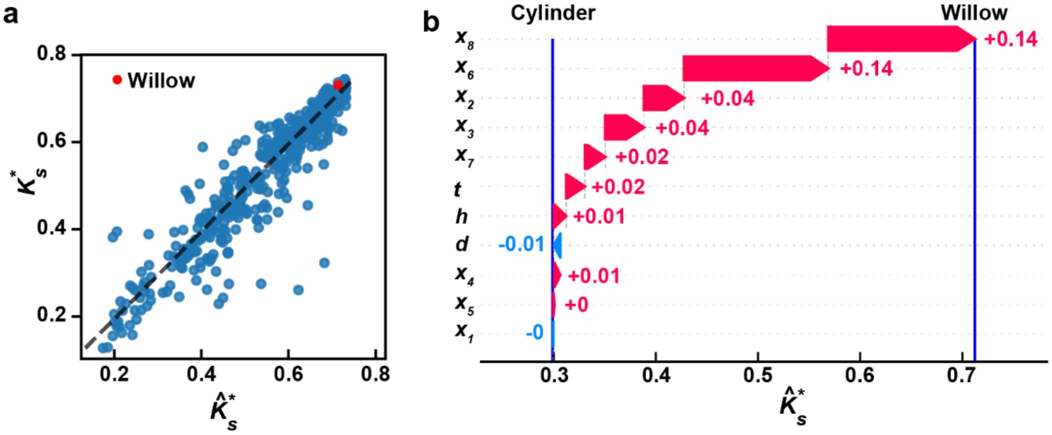


**Fig. S15 | Shapley additive explanations (SHAP) analysis of Willow as the superlative design.** **a**, Parity plot of the neural network built on all data taken using PLA with Willow highlighted. **b**, SHAP waterfall plot for the Willow design tested in PLA relative to a PLA cylindrical shell with the same height, diameter, and thickness. These values show the cumulative effect of positive (red) or negative (blue) contributions of individual feature values to model predictions.

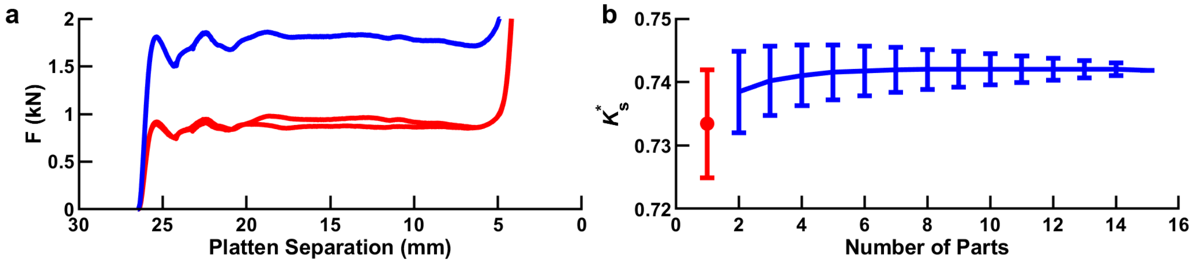


**Fig. S16 | Simulation of efficiency for combinations of tested willow components. a,** Two or more experimental *F*-separation curves (red) can be combined to create a simulated test of in-plane tiled components (blue). Curves are added in platen separation space to account for possible variations in component height. **b,** Mean and one standard deviation in $K_s^*$ for original components (red) and combined prediction (blue) for varying number of components.

While this study focused on energy absorbing efficiency, both the toughness per unit mass (Figure S17a) and toughness per unit volume (Figures S17b) are interesting properties to examine. For both metrics, performance is correlated with both material strength and component effective medium density. Despite this, the toughness of a component is unbounded in compression due to the densification of most structures. To illustrate this issue, consider both the Willow and Iroko structures from Figure 3. By measuring the specific toughness below a given $\sigma_t$, it demonstrates that most of the energy is absorbed before the critical stress, but that $U_v$ continues to increase as $\sigma_t$

increases. This makes it difficult to compare toughness tested on instruments that have different force limits unless the performance is bounded by some force or stress threshold. For this reason, employing $K_s$ allows not only comparison across different materials, but also mitigates different testing limitations based on machine capabilities or user choices.

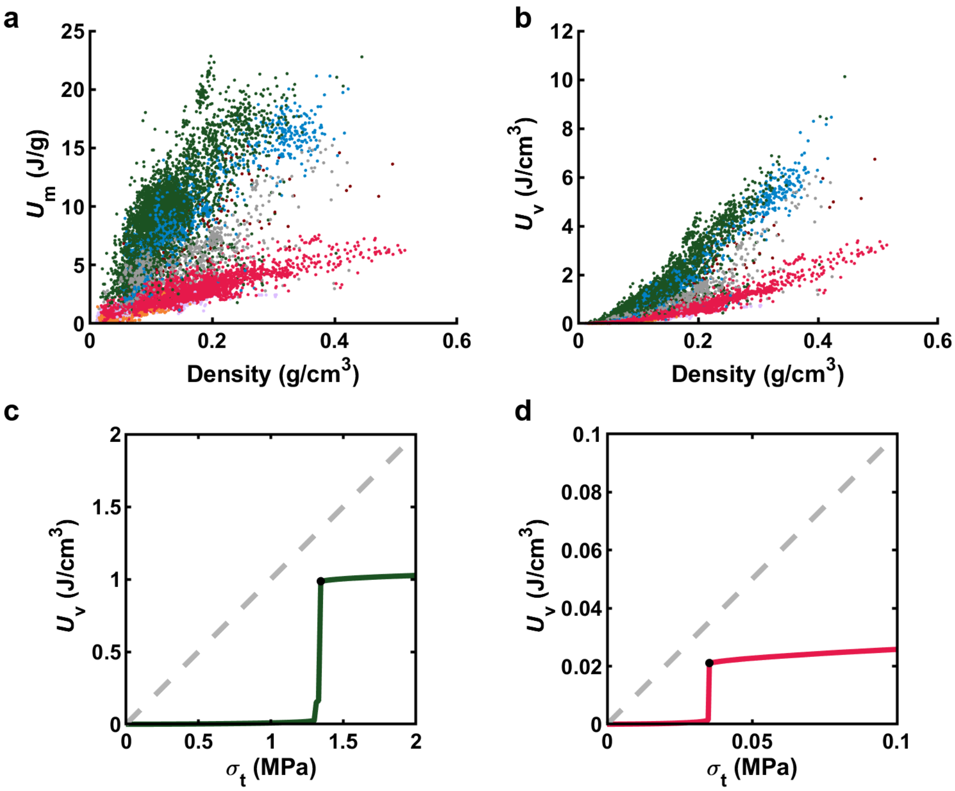


**Fig. S17 | Examining toughness metrics beyond energy absorbing efficiency. a,** Toughness per mass $U_m$ vs. component effective medium density for all successful experiments. The color of each dot corresponds to the material used, as designated in Figure S4. **b,** Toughness per volume $U_v$ vs. component effective medium density for all successful experiments. **c,** $U_v$ vs $\sigma_t$ for the original Willow component with the point representing $K_s^*$ marked by a black dot and the parity line showing the maximum possible $U_v$. **d,** $U_v$ vs $\sigma_t$ for the original Iroko component.

## Supplemental References

1. Noack, M. & Zwart, P. Computational Strategies to Increase Efficiency of Gaussian-Process-Driven Autonomous Experiments. in *2019 IEEE/ACM 1st Annual Workshop on Large-scale Experiment-in-the-Loop Computing (XLOOP)* 1–7 (IEEE, 2019).

2. Szegedy, C. *et al.* Going deeper with convolutions. in *Proceedings of the IEEE conference on computer vision and pattern recognition* 1–9 (2015).

3. Snapp, K. L., Gongora, A. E. & Brown, K. A. Increasing throughput in fused deposition modeling by modulating bed temperature. *J. Manuf. Sci. Eng. Trans. ASME* **143**, 094502 (2021).

4. Lundberg, S. M. & Lee, S. I. A unified approach to interpreting model predictions.

*Advances in Neural Information Processing Systems* vol. 30 (2017).

5. Nair, V. & Hinton, G. E. Rectified linear units improve restricted boltzmann machines. in *Proceedings of the 27th International Conference on International Conference on Machine Learning* 807–814 (Omnipress, 2010).

6. Kingma, D. P. & Ba, J. Adam: A method for stochastic optimization. in *3rd International Conference on Learning Representations, ICLR 2015 - Conference Track Proceedings* (arXiv, 2015).
